# Supplementary material for: A novel trajectory learning method for robotic arms based on Gaussian Mixture Model and k-value selection algorithm
Source: PLoS One. 2025 Feb 14;20(2):e0318403. doi: 10.1371/journal.pone.0318403 (PMC11828358; doi:10.1371/journal.pone.0318403)
Supplement: S1 File — (DOCX) [file pone.0318403.s001.docx]

**Supporting information (Schedule)**

S1 Table. Detailed results of MAE for DMP, GMM, and GMM-KVS (simulation experiment).

| Method | MAE | | | Average MAE |
| --- | --- | --- | --- | --- |
|  | System 1 | System 2 | System 3 |  |
| DMP | 0.0314 | 0.0819 | 0.0281 | 0.0570 |
|  | 0.0137 | 0.0912 | 0.0367 |  |
|  | 0.0084 | 0.0902 | 0.0614 |  |
|  | 0.0943 | 0.0655 | 0.0444 |  |
|  | 0.0719 | 0.0884 | 0.0273 |  |
|  | 0.0418 | 0.0936 | 0.0566 |  |
| GMM | 0.0176 | 0.0186 | 0.0028 | 0.0242 |
|  | 0.0115 | 0.0235 | 0.0050 |  |
|  | 0.0054 | 0.0497 | 0.0067 |  |
|  | 0.0662 | 0.0247 | 0.0182 |  |
|  | 0.0524 | 0.0336 | 0.0123 |  |
|  | 0.0416 | 0.0350 | 0.0113 |  |
| GMM-KVS | 0.0093 | 0.0077 | 0.0023 | 0.0159 |
|  | 0.0100 | 0.0081 | 0.0045 |  |
|  | 0.0048 | 0.0249 | 0.0057 |  |
|  | 0.0518 | 0.0164 | 0.0169 |  |
|  | 0.0461 | 0.0210 | 0.0112 |  |
|  | 0.0175 | 0.0171 | 0.0105 |  |

S2 Table. Detailed results of RMSE for DMP, GMM, and GMM-KVS (simulation experiment).

| Method | RMSE | | | Average RMSE |
| --- | --- | --- | --- | --- |
|  | System 1 | System 2 | System 3 |  |
| DMP | 0.0345 | 0.1034 | 0.0307 | 0.0679 |
|  | 0.0147 | 0.1115 | 0.0455 |  |
|  | 0.0099 | 0.1070 | 0.0671 |  |
|  | 0.1119 | 0.0836 | 0.0513 |  |
|  | 0.0843 | 0.1127 | 0.0361 |  |
|  | 0.0478 | 0.1059 | 0.0638 |  |
| GMM | 0.0189 | 0.0211 | 0.0035 | 0.0274 |
|  | 0.0136 | 0.0267 | 0.0058 |  |
|  | 0.0062 | 0.0571 | 0.0076 |  |
|  | 0.0795 | 0.0255 | 0.0196 |  |
|  | 0.0619 | 0.0355 | 0.0138 |  |
|  | 0.0462 | 0.0376 | 0.0128 |  |
| GMM-KVS | 0.0109 | 0.0084 | 0.0032 | 0.0184 |
|  | 0.0114 | 0.0089 | 0.0051 |  |
|  | 0.0058 | 0.0282 | 0.0065 |  |
|  | 0.0621 | 0.0174 | 0.0183 |  |
|  | 0.0589 | 0.0220 | 0.0126 |  |
|  | 0.0210 | 0.0184 | 0.0120 |  |

S3 Table. Detailed results of MAE and RMSE for DMP, GMM, and GMM-KVS (robotic arm experiment).

| Method | MAE | Average MAE | RMSE | Average RMSE |
| --- | --- | --- | --- | --- |
| DMP | 7.7973 | 8.4633 | 9.1280 | 10.0753 |
|  | 5.1625 |  | 6.6734 |  |
|  | 12.4300 |  | 14.4245 |  |
| GMM | 5.5070 | 6.6747 | 6.4574 | 7.7971 |
|  | 3.8079 |  | 4.4576 |  |
|  | 10.7090 |  | 12.4764 |  |
| GMM-KVS | 4.6264 | 5.3859 | 5.3844 | 6.2729 |
|  | 2.9434 |  | 3.4698 |  |
|  | 8.5880 |  | 9.9646 |  |
