# Supplementary material for: A novel trajectory learning method for robotic arms based on Gaussian Mixture Model and k-value selection algorithm
Source: PLoS One. 2025 Feb 14;20(2):e0318403. doi: 10.1371/journal.pone.0318403 (PMC11828358; doi:10.1371/journal.pone.0318403)
Supplement: Attachment — Submitted filename: OSQ PEER REVIEWER 2.pdf [file pone.0318403.s002.pdf]

Peer Review of "A Novel Trajectory Learning Method for Robotic Arms Based on Gaussian Mixture Model and k-Value Selection Algorithm"

General Assessment

The manuscript introduces an innovative trajectory learning approach for robotic arms, leveraging the Gaussian Mixture Model (GMM) combined with a k-value selection algorithm. This study addresses a significant challenge in trajectory learning—selecting the optimal k-value for GMM, which impacts trajectory accuracy. By combining GMM with a refined elbow method, this work offers a methodologically rigorous solution with strong experimental support. However, certain areas require clarification and elaboration, particularly in methodology and experimental comparison.

Major Comments

1. Innovation and Significance

Strengths:

The proposed integration of GMM with a k-value selection algorithm represents a well-motivated solution to improve trajectory learning in robotic arms. The study's relevance is emphasized by the improvements observed over traditional GMM and Dynamic Movement Primitives (DMP) methods. This enhancement is particularly impactful for industrial robotics, where accurate trajectory learning significantly affects application efficiency.

Suggestions for Enhancement:

Expand on the novelty of the k-value selection process, detailing the improvements and unique contributions compared to existing trajectory planning techniques using GMM. Highlight how optimizing the k-value selection can improve trajectory precision, making it advantageous for applications in industrial assembly and logistics.

2. Methodology

Strengths:

The manuscript provides a well-structured outline of the proposed method, particularly the k-value selection, initialization of the GMM via k-means clustering, and training using Expectation-Maximization (EM).

Suggested Improvements:

Detailed Algorithm Explanation: The manuscript introduces complex mathematical adjustments to the elbow method (Equations 4–7). Additional clarification, perhaps through a flowchart, would enhance readability.

Explain the significance of terms like the "scaling weight θ" and its practical implications in avoiding exponential overflow.

Comparison with Traditional Methods:

Integrate a more comprehensive comparison of the improved elbow method against alternative selection criteria (e.g., Bayesian Information Criterion, Akaike Information Criterion) to clarify its applicability and advantages. Additionally, suggest comparisons with other common trajectory learning techniques, such as deep learning or demonstration-based learning, to highlight scenarios where the proposed method may excel.

3. Experimental Validation

Strengths:

The experimental design is robust, covering both simulation and real-world scenarios using a 6-DOF robotic arm. The results in both the Mean Absolute Error (MAE) and Root Mean Square Error (RMSE) provide quantitative support for the superiority of the proposed method.

Areas for Improvement:

Clarification of Experimental Setup: Offer more details about the data acquisition in the physical experiment, including how many trials were averaged and the variability observed across trials.

Figures and Graphs:

Some figures (e.g., Figs. 5 and 6) could be enhanced by labeling critical points, such as the selected optimal k-values. Color-coding or special markers for these points could improve understanding of the selection process.

Generalization Across Systems:

The manuscript's choice of three nonlinear dynamical systems is appropriate for assessing the model's generalization. However, it would be beneficial to discuss the applicability of the proposed method to other domains or types of robotic systems beyond the specified 6-DOF arm. Consider suggesting future studies to apply this technique to mobile robots or drones to assess its generalizability.

4. Discussion and Practical Applications

Strengths: The study effectively acknowledges its practical applications, specifically in dynamically adaptive robotic movement.

Suggested Additions:

- *Broader Applications: Discuss additional practical contexts where this method could be applied (e.g., industrial assembly lines, autonomous logistics robots) to underscore its impact.*
- *Limitations: Elaborate on potential limitations, such as computational requirements for larger datasets, and suggest future work directions to address these limitations.*

5. Statistical Analysis

Strengths:

The use of error metrics (MAE, RMSE) is appropriate and helps quantify the performance.

Recommendations:

Consider including confidence intervals for MAE and RMSE to provide a more robust statistical basis. Additionally, clarifying how significance was assessed would strengthen the reliability of the findings.

Minor Comments

1. Language and Style:

Some technical phrases could be simplified for broader readability, especially for an interdisciplinary audience. For example, phrases like "iteratively calculating the SSE for different k-values and plotting the line graph" could be rephrased for smoother flow.

2. Figures and Tables:

Several figures lack detailed captions, such as Fig. 6. Captions should describe key findings or critical points, especially where the proposed algorithm surpasses existing methods.

3. Citations and Literature Review:

Consider adding recent studies that explore adaptive GMM methods in other fields to bolster the background section.

Ethical and Technical Considerations

Ethical Standards: The study adheres to ethical standards, with clear disclosure of no competing interests and no ethical issues arising from the methods used.

Technical Soundness: The manuscript is technically sound, though improved explanations for mathematical adjustments and the k-value selection algorithm would enhance comprehensibility for readers.

Recommendation

The research demonstrates notable technical rigor and offers a valuable contribution to the field of robotic trajectory learning. Most of the recommended changes focus on improving clarity, methodological depth, and robustness of the statistical validation.

Verdict: **Minor revisions** *to improve clarity and expand on the limitations and broader applicability. The manuscript will be suitable for publication following these revisions.*

Summary of Key Points

The study introduces a novel k-value selection algorithm within GMM-based robotic trajectory learning.Experimental validation supports the model's performance, but a few methodological and interpretive clarifications are recommended. With minor revisions, the manuscript presents a well-substantiated, impactful approach for enhanced robotic trajectory learning.

GOOD LUCK
