# Supplementary material for: A novel trajectory learning method for robotic arms based on Gaussian Mixture Model and k-value selection algorithm
Source: PLoS One. 2025 Feb 14;20(2):e0318403. doi: 10.1371/journal.pone.0318403 (PMC11828358; doi:10.1371/journal.pone.0318403)
Supplement: Attachment — Submitted filename: Response to Reviewers.docx [file pone.0318403.s004.docx]

**Response to Academic Editor**

Thank you for submitting your manuscript to PLOS ONE. After careful consideration, we feel that it has merit but does not fully meet PLOS ONE’s publication criteria as it currently stands. Therefore, we invite you to submit a revised version of the manuscript that addresses the points raised during the review process.

According to the feedback from the reviewers, there are still several problems that need to be improved:

1) The abstract and conclusion need further refinement. Meanwhile, a discussion on the application and limitation is suggested.

2) The novelty of this manuscript should be clearly clarified.

3) Most of the figures in this manuscript is not clear and a further adjustment is expected.

4) More details about the methodology and experimental setup are missing.

Thank you for your constructive feedback. We have completed the revisions, and the changes in the revised manuscript are highlighted in blue text. We have also addressed the comments from both the reviewers and the editorial team.

**Response:** 1) We have revised the abstract and conclusion to clarify the significance of our findings, including the scientific gap, the novelty of our method, and key results such as the 15% improvement in trajectory accuracy. We also added a discussion of the practical applications of our method and its limitations.

2) The novelty of our work is now more clearly explained. Specifically, we highlight the integration of the improved k-value selection algorithm with the Gaussian Mixture Model, which significantly enhances trajectory learning accuracy compared to traditional methods.

3) We have enhanced the resolution of all figures and ensured that the text within the figures is clear and legible, meeting the publication standards.

4) We have expanded the methodology and experimental setup sections to provide more comprehensive details on the GMM-KVS methods, as well as the experimental environment and data collection process.

We have completed the revisions to the manuscript. In addition, we have provided responses to the comments from the two reviewers included in the email, as well as to the peer review feedback in the attached document.

**Response to Reviewer 1**

Sincerely thank you for your comments. Next will be the responses to these comments one by one.

1. The abstract need to be rewritten in more professional way, mention the gap of the science and the importance and the logic of this study, the methodology more detailed and the most important results such as quantities or percentage of improvement.

**Response:** We have rewritten the abstract to clearly highlight the scientific gap addressed by the study, the importance of the research, and the logical flow of the work. We also provided more details on the methodology and emphasized the key results, including the over 15% improvement in trajectory accuracy. The revised abstract is as follows:

In the field of robotic arm trajectory imitation learning, Gaussian Mixture Models are widely used for their ability to capture the characteristics of complex trajectories. However, one major challenge in utilizing these models lies in the initialization process, particularly in determining the number of Gaussian kernels, or the k-value. The choice of the k-value significantly impacts the model's performance, and traditional methods, such as random selection or selection based on empirical knowledge, often lead to suboptimal outcomes. To address this challenge, this paper proposes a novel trajectory learning method for robotic arms that combines Gaussian Mixture Model with a k-value selection algorithm. The proposed approach leverages the principles of the elbow method along with the properties of exponential functions, correction terms, and weight adjustments to accurately determine the optimal k-value. Next, k-means clustering is applied with the optimal k-value to initialize the parameters of the Gaussian Mixture Model, which are then refined and trained through the Expectation-Maximization algorithm. The resulting model parameters are then employed in Gaussian Mixture Regression to generate the robotic arm trajectories. The effectiveness of the proposed method is validated through both simulation experiments with two-dimensional theoretical nonlinear dynamic systems and physical experiments with actual robotic arm data. Experimental results demonstrate that, compared to the traditional Gaussian Mixture Model approach, the proposed method improves trajectory accuracy by more than 15%, as shown by reductions in both the Mean Absolute Error and the Root Mean Square Error. These results highlight that the proposed method significantly enhances the accuracy and efficiency of robotic arm trajectory generation, providing a promising solution for improving robotic manipulation tasks.

2. The conclusion needs to cover the most significant achievements, including some comparison in percentage or another professional way.

**Response:** The conclusion has been revised to more clearly highlight the significant achievements of the study, with a professional comparison of the results, expressed in percentage terms, to demonstrate the improvements over traditional methods. A part of the revised conclusion is as follows:

This paper presents a novel robotic arm trajectory learning method based on Gaussian Mixture Models combined with a k-value selection algorithm. By optimizing the initialization process through precise determination of the number of Gaussian kernels, the method avoids selection biases and significantly improves trajectory learning performance. Experimental results demonstrate that the proposed method enhances trajectory accuracy by over 15%, offering substantial improvements in precision and reliability compared to traditional approaches. These advancements contribute to the operational capability of robotic arms, particularly in complex and dynamic environments. The proposed method is not limited to a specific type or model of robotic arm, making it highly adaptable and suitable for a wide range of applications. It can be applied to various robotic systems, such as industrial assembly lines and autonomous logistics robots, where high precision and adaptability are essential for efficient operation across diverse tasks and environments.

3. Considering to compare tables 1 and 2 data would provide some more useful information.

**Response:** We have added a comparative analysis of the data in Tables 1 and 2, emphasizing the performance differences between the methods in both the simulation and robotic arm experiments. This analysis provides a clearer interpretation of the results, highlighting that the GMM-KVS method shows greater improvements in the simulation experiment, where conditions are more controlled. In the robotic arm experiment, while the improvements are somewhat smaller, the method still demonstrates strong performance. The modified section is presented below:

When comparing the results in Table 2 with those in Table 1, it is evident that the GMM-KVS method consistently outperforms both the GMM and DMP methods, showing significant improvements in both the simulation and robotic arm experiments. Notably, the GMM-KVS method demonstrates a greater improvement in the simulation experiment compared to the robotic arm experiment. This discrepancy can be attributed to the more controlled and idealized conditions in the simulation, where factors such as noise and external disturbances are minimized. In contrast, the physical robotic arm experiment involves additional complexities, which may reduce the method's effectiveness. Despite these challenges, the GMM-KVS method still provides substantial improvements in the robotic arm experiment, highlighting its robustness in real-world applications.

4. Consider reproduce the unclear pictures. Please revise all figures and make sure texts also have a good resolution.

**Response:** Thank you for your valuable feedback. We have reviewed all figures in the manuscript and ensured that the text in the figures is of high resolution and clearly readable.

Sincerely thank you for your efforts in reviewing our manuscript. All the comments are valuable and helpful for improving the quality of our manuscript.

**Response to Reviewer 2**

The manuscript presents a novel approach for robotic arm trajectory learning using a Gaussian Mixture Model (GMM) with a k-value selection algorithm. This innovative combination addresses key challenges in trajectory accuracy. The research is technically sound and well-supported by experiments, but several improvements are suggested:

Sincerely thank you for your comments. Next will be the responses to these comments one by one.

1. Clarify the Novelty: Expand on the unique aspects of the k-value selection method compared to existing approaches.

**Response:** Thank you for your comments. To address your suggestion, we have expanded the discussion on the novelty of our k-value selection method. Traditional approaches, such as random selection or empirical estimation, often result in suboptimal k-values, which can significantly impact the performance of Gaussian Mixture Models in trajectory learning. Furthermore, while the elbow method is commonly used for k-value selection, it has notable limitations, particularly in cases where the "elbow point" is not well-defined, leading to ambiguities in determining the optimal k-value.

In contrast, our proposed method improves upon these traditional techniques by introducing an enhanced k-value selection algorithm that integrates exponential function characteristics, correction terms, and weight adjustments. This approach allows for a more accurate and robust determination of the optimal k-value, which is essential for ensuring precise model initialization and, consequently, better trajectory learning results. By refining the k-value selection process, our method significantly enhances trajectory accuracy, making it particularly suitable for complex and dynamic environments, such as industrial assembly lines and autonomous logistics robots.

2. Methodology Details: Add further explanation for the mathematical steps and consider comparisons with other k-selection methods.

**Response:** Thank you for your valuable feedback. In response, we have clarified the explanation of the k-value selection algorithm, particularly the mathematical adjustments introduced to the elbow method in Equations (4) to (7). We have elaborated on the role of the scaling weight (θ), which prevents the "exponential explosion" phenomenon that can occur when points are far from their group’s centroid. By adjusting the exponential term, θ moderates the influence of extreme values, ensuring they do not distort the final k-value selection. This adjustment enhances the robustness and accuracy of the k-value determination, making the algorithm more reliable for practical applications.

Additionally, we have expanded the comparison of our method with traditional selection criteria, such as the Bayesian Information Criterion (BIC). While the BIC is effective for model selection, it may not always perform well when the clustering structure is ambiguous. Our method addresses this limitation by improving both robustness and precision in k-value determination, ensuring more accurate results. Furthermore, we compared our method to deep learning approaches, which typically require large datasets and significant computational resources. In contrast, our method, based on unsupervised learning, can achieve high accuracy with smaller datasets, making it more efficient and practical in situations where data is limited or expensive to acquire.

3. Experimental Clarity: Provide more details on the physical experiment setup and improve figure labeling.

**Response:** Thank you for your valuable suggestion. We have supplemented the manuscript with additional information regarding the data acquisition process in the physical experiment. Specifically, to verify the effectiveness of the proposed method in real-world scenarios, experiments were conducted using a 6-DOF robotic arm, AUBO-C5. The process involved the demonstrator picking up an object and dragging the end-effector of the robotic arm to the specified position. This process was repeated across 9 trials, with each trial starting from a different initial position, but all trials aimed to reach the same target point. While the trajectories were intended to be as similar as possible in shape, slight variations and small oscillations were observed due to factors such as minor human inconsistencies and the inherent flexibility of the robotic system. These recorded trajectories were used as input for the imitation learning process.

Additionally, we have updated Figures 6 and 13(B) to clearly label the critical points. Specifically, we have marked the elbow points, which correspond to the optimal k-values, to highlight these key locations. These updates aim to improve the understanding of the methodology and enhance the overall readability of the figures.

Fig 6. Line chart plotted using the k-value selection algorithm: (A) system 1; (B) system 2; (C) system 3.

Fig 13. Line charts of the elbow method and the k-value selection algorithm: (A) elbow method; (B) k-value selection algorithm.

4. Applications and Limitations: Discuss broader applications and potential limitations of the method.

**Response:** Thank you for your suggestion to further discuss the broader applications and potential limitations of the proposed method. In response, we have expanded the conclusion to highlight the wide applicability of our approach. Since the method focuses on robotic arm trajectory learning, it can be applied to a variety of robotic systems, irrespective of their model or design. This includes, but is not limited to, industrial assembly lines and autonomous logistics robots. The versatility of the approach makes it a promising solution for improving robotic systems in diverse real-world environments that require precise motion control and adaptability.

Regarding limitations, we acknowledge that the performance of the method is dependent on the quality of the initial trajectory data. Noisy, incomplete, or inconsistent data could negatively impact the learning process and accuracy of the generated trajectories. Additionally, as the method involves iterative processes and the selection of model parameters, it can become computationally intensive when working with large datasets or in real-time applications. In the future, we plan to focus on improving the robustness of the method to noise and enhancing its scalability, so it can better handle larger datasets and operate efficiently in real-time systems.

5. Statistical Validation: Include confidence intervals to enhance the statistical robustness of the findings.

**Response:** Thank you for your valuable suggestion. In response, we have included 95% confidence intervals for both the Mean Absolute Error (MAE) and Root Mean Square Error (RMSE) to provide a more robust statistical basis for the findings. The confidence intervals for each method (DMP, GMM, and GMM-KVS) are provided in the revised manuscript. Specifically, for MAE, the confidence intervals for the DMP, GMM, and GMM-KVS methods are [0.0436, 0.0705], [0.0157, 0.0327], and [0.0097, 0.0221], respectively. For RMSE, the confidence intervals are [0.0515, 0.0843], [0.0174, 0.0374], and [0.0107, 0.0261], respectively.

We also clarify how significance was assessed in the manuscript by comparing the confidence intervals of each method. The narrower confidence intervals for the GMM-KVS method indicate more consistent and reliable results, reinforcing the statistical robustness of the findings. We believe that these additions strengthen the reliability of our conclusions, as they provide a more comprehensive statistical evaluation of the performance improvements.

Sincerely thank you for your efforts in reviewing our manuscript. All the comments are valuable and helpful for improving the quality of our manuscript.

**Response to Peer Review**

**General Assessment**

The manuscript introduces an innovative trajectory learning approach for robotic arms, leveraging the Gaussian Mixture Model (GMM) combined with a k-value selection algorithm. This study addresses a significant challenge in trajectory learning—selecting the optimal k-value for GMM, which impacts trajectory accuracy. By combining GMM with a refined elbow method, this work offers a methodologically rigorous solution with strong experimental support. However, certain areas require clarification and elaboration, particularly in methodology and experimental comparison.

Sincerely thank you for your comments. Next will be the responses to these comments one by one.

**Major Comments**

**1. Innovation and Significance**

Expand on the novelty of the k-value selection process, detailing the improvements and unique contributions compared to existing trajectory planning techniques using GMM. Highlight how optimizing the k-value selection can improve trajectory precision, making it advantageous for applications in industrial assembly and logistics.

**Response:** Thank you for your valuable feedback. In response to your suggestion to expand on the novelty of the k-value selection process, we have revised the manuscript to provide more detailed explanations about the improvements and unique contributions of our method. Specifically, the k-value selection algorithm introduced in this paper optimizes the determination of the number of Gaussian kernels, which is a critical step in Gaussian Mixture Model-based trajectory learning. Traditional methods, such as random selection or empirical estimation, often lead to suboptimal k-values, negatively impacting trajectory accuracy. Additionally, the commonly used elbow method for k-value selection has limitations, particularly when the "elbow point" is not clearly defined, which can result in inaccurate k-value choices. To address this, we proposed an improved version of the elbow method, which incorporates exponential functions, correction terms, and weight adjustments to more accurately select the optimal k-value.

This optimization significantly enhances trajectory precision, making the method particularly advantageous for real-world applications like industrial assembly lines and autonomous logistics robots. In these applications, precise and adaptable trajectories are crucial for efficient and reliable operation. By improving both trajectory accuracy and model robustness, our approach ensures better performance in dynamic and complex environments.

**2. Methodology**

Detailed Algorithm Explanation: The manuscript introduces complex mathematical adjustments to the elbow method (Equations 4–7). Additional clarification, perhaps through a flowchart, would enhance readability. Explain the significance of terms like the “scaling weight θ” and its practical implications in avoiding exponential overflow.

**Response:** Thank you for your suggestion. In response, we have further clarified the explanation of the algorithm, particularly focusing on the mathematical adjustments introduced to the elbow method in Equations (4) to (7). We have elaborated on the key concepts, including the scaling weight (θ) and its practical role in moderating the influence of extreme values during the k-value selection process.

The scaling weight (θ) is introduced to prevent the "exponential explosion" phenomenon that can occur when some points are far from their group’s centroid. By adjusting the exponential term, θ ensures that extreme distances do not overly distort the final k-value selection, thus maintaining the stability and robustness of the algorithm. This adjustment enhances the accuracy of the k-value determination, making the method more reliable in practical applications.

Comparison with Traditional Methods: Integrate a more comprehensive comparison of the improved elbow method against alternative selection criteria (e.g., Bayesian Information Criterion, Akaike Information Criterion) to clarify its applicability and advantages. Additionally, suggest comparisons with other common trajectory learning techniques, such as deep learning or demonstration-based learning, to highlight scenarios where the proposed method may excel.

**Response:** Thank you for your valuable suggestion. In response, we have revised the manuscript to include a more comprehensive comparison of our proposed k-value selection algorithm against traditional selection criteria, such as the Bayesian Information Criterion (BIC). While the BIC is widely used for model selection, it may not always perform well when the underlying clustering structure is unclear, potentially leading to suboptimal results. Our method, in contrast, improves the k-value selection process by enhancing its robustness and accuracy. This comparison highlights the advantages of our approach in accurately determining the optimal number of clusters for robotic arm trajectory learning.

Additionally, we compared our method with deep learning approaches, which generally require large datasets and significant computational resources. In contrast, the GMM-KVS method is based on unsupervised learning and can achieve high accuracy with smaller datasets. This makes our approach more efficient and practical, particularly in scenarios where data is limited or expensive to acquire. The revised manuscript reflects these comparisons and clarifies the applicability and advantages of the proposed method in various trajectory learning contexts.

**3. Experimental Validation**

Clarification of Experimental Setup: Offer more details about the data acquisition in the physical experiment, including how many trials were averaged and the variability observed across trials.

**Response:** Thank you for your valuable suggestion. In response, we have supplemented the manuscript with more details regarding the data acquisition process in the physical experiment. The revisions are as follows:

To verify the effectiveness of the proposed method in real-world scenarios, experiments were conducted using a 6-DOF robotic arm, AUBO-C5. After picking up an object, the demonstrator drags the end-effector of the robotic arm to the specified position, as shown in Fig 11. The process was repeated 9 times, with each trial starting from a different initial position, but all trials aimed to reach the same target point. Although the trajectories were intended to be similar in shape, slight variations and small oscillations were inevitably observed due to factors such as minor human inconsistencies and the inherent flexibility of the robotic system. The trajectories were recorded and used as input for the imitation learning process, as shown in Fig. 12.

Figures and Graphs: Some figures (e.g., Figs. 5 and 6) could be enhanced by labeling critical points, such as the selected optimal k-values. Color-coding or special markers for these points could improve understanding of the selection process.

**Response:** Thank you for your valuable suggestion. In response, we have updated Figures 6 and 13(B) to clearly label the critical points. Specifically, we have marked the elbow points, which correspond to the optimal k-values, to highlight these key locations. We believe these changes will provide a better understanding of the methodology and improve the overall readability of the figures.

Fig 6. Line chart plotted using the k-value selection algorithm: (A) system 1; (B) system 2; (C) system 3.

Fig 13. Line charts of the elbow method and the k-value selection algorithm: (A) elbow method; (B) k-value selection algorithm.

Generalization Across Systems: The manuscript’s choice of three nonlinear dynamical systems is appropriate for assessing the model’s generalization. However, it would be beneficial to discuss the applicability of the proposed method to other domains or types of robotic systems beyond the specified 6-DOF arm. Consider suggesting future studies to apply this technique to mobile robots or drones to assess its generalizability.

**Response:** Thank you for your valuable suggestion. Although this experiment uses a specific 6-DOF robotic arm, the proposed method is based on trajectory generation and learning, which makes it applicable to a wide variety of robotic systems. The method is not constrained to a specific manipulator model, and its principles can be extended to other robotic platforms, including mobile robots and drones, that rely on trajectory-based control.

We have also highlighted that future studies could explore the applicability of the proposed technique to other domains, such as mobile robotics and drones, in order to assess its generalizability across different types of robotic systems with varying dynamics.

**4. Discussion and Practical Applications**

Broader Applications: Discuss additional practical contexts where this method could be applied (e.g., industrial assembly lines, autonomous logistics robots) to underscore its impact.

**Response:** Thank you for your suggestion to discuss the broader applications of the proposed method. We have expanded the conclusion to emphasize the versatility of our approach. Since our method is based on robotic arm trajectory learning, it is not limited to a specific type or model of robotic arm, making it highly adaptable for various robotic systems. This includes applications such as industrial assembly lines and autonomous logistics robots, where precise trajectory learning and adaptability are critical across different tasks and environments.

Limitations: Elaborate on potential limitations, such as computational requirements for larger datasets, and suggest future work directions to address these limitations.

**Response:** We have also addressed the limitations of the proposed method in the revised conclusion. Specifically, we have noted that the performance of the method is dependent on the quality of the initial trajectory data, and that challenges may arise with noisy or incomplete data. Additionally, we acknowledge that the computational complexity could become a bottleneck when applied to larger datasets or real-time systems. To address these limitations, future work will focus on improving the robustness of the method to noise and enhancing its scalability. We also plan to explore techniques for optimizing computational efficiency, which will be crucial for enabling the method to handle large-scale datasets and real-time applications.

**5. Statistical Analysis**

Consider including confidence intervals for MAE and RMSE to provide a more robust statistical basis. Additionally, clarifying how significance was assessed would strengthen the reliability of the findings.

**Response:** Thank you for your valuable suggestion. In response, we have included 95% confidence intervals for both the Mean Absolute Error (MAE) and Root Mean Square Error (RMSE) to provide a more robust statistical basis for the findings. The confidence intervals for each method (DMP, GMM, and GMM-KVS) are provided in the revised manuscript. Specifically, for MAE, the confidence intervals for the DMP, GMM, and GMM-KVS methods are [0.0436, 0.0705], [0.0157, 0.0327], and [0.0097, 0.0221], respectively. For RMSE, the confidence intervals are [0.0515, 0.0843], [0.0174, 0.0374], and [0.0107, 0.0261], respectively.

We also clarify how significance was assessed in the manuscript by comparing the confidence intervals of each method. The narrower confidence intervals for the GMM-KVS method indicate more consistent and reliable results, reinforcing the statistical robustness of the findings. We believe that these additions strengthen the reliability of our conclusions, as they provide a more comprehensive statistical evaluation of the performance improvements.

**Minor Comments**

**1. Language and Style**

Some technical phrases could be simplified for broader readability, especially for an interdisciplinary audience. For example, phrases like "iteratively calculating the SSE for different k-values and plotting the line graph" could be rephrased for smoother flow.

**Response:** Thank you for your valuable suggestion. In response, we have simplified certain technical phrases for broader readability, particularly for an interdisciplinary audience. For example, we have revised the phrase "iteratively calculating the SSE for different k-values and plotting the line graph" to "calculating the SSE for different k-values and plotting the results." Additionally, we have reviewed and revised other sections of the manuscript to ensure smoother flow and accessibility for a wider range of readers.

**2. Figures and Tables**

Several figures lack detailed captions, such as Fig. 6. Captions should describe key findings or critical points, especially where the proposed algorithm surpasses existing methods.

**Response:** Thank you for your valuable suggestion. In response, we have added more detailed descriptions to the figures, including Fig. 6, to better explain the key findings and highlight the critical points, particularly where the proposed algorithm surpasses existing methods. The revised description for Fig. 6 is as follows:

As shown in Figs 5 and 6, when using the elbow method for k-value selection, it is not possible to intuitively determine the optimal k-value from the graphs for Systems 1-3, as there is no clear "elbow point" in the figures. However, by applying the k-value selection algorithm proposed in this paper, the limitations of the elbow method can be overcome. With this algorithm, the optimal k-value can be intuitively identified from the line graph, where the "elbow point" is clearly marked (as indicated by the red points). The optimal k-values for Systems 1, 2, and 3 are 7, 6, and 6, respectively. This demonstrates that the k-value selection algorithm used in this paper has a broader applicability compared to the elbow method and is significant for the final trajectory learning results.

**3. Citations and Literature Review**

Consider adding recent studies that explore adaptive GMM methods in other fields to bolster the background section.

**Response:** Thank you for your valuable suggestion. In response, we have added a recent study on the GMM method to support the background section. The specific content is as follows:

Jia [21], addressing the challenge of robot arm trajectory planning in unstructured environments, proposed a method that utilizes the Gaussian Mixture Model and Gaussian Mixture Regression to generate ideal primitive trajectory actions, significantly improving the adaptability and generalization performance of robotic arms.

[21]	Jia X, Zhao B, Liu J, Zhang S. A trajectory planning method for robotic arms based on improved dynamic motion primitives. Industrial Robot. 2024;51(5):847-856.

Sincerely thank you for your efforts in reviewing our manuscript. All the comments are valuable and helpful for improving the quality of our manuscript.
